# Editorial: Polycystic ovary syndrome: mechanism and management—volume II

**DOI:** 10.3389/fendo.2023.1247679

**Published:** 2023-07-31

**Authors:** Yishu Wang, Peter Leung, Rong Li, Yanting Wu, Hefeng Huang

**Affiliations:** ^1^ International Peace Maternity and Child Health Hospital, School of Medicine, Shanghai Jiao Tong University, Shanghai, China; ^2^ Research Units of Embryo Original Diseases, Chinese Academy of Medical Sciences, Shanghai, China; ^3^ Obstetrics and Gynecology Hospital, Institute of Reproduction and Development, Fudan University, Shanghai, China; ^4^ Department of Obstetrics and Gynaecology, BC Children’s Hospital Research Institute, University of British Columbia, Vancouver, BC, Canada; ^5^ Centre for Reproductive Medicine, Department of Obstetrics and Gynecology, Peking University Third Hospital, Beijing, China; ^6^ National Clinical Research Centre for Obstetrics and Gynecology, Beijing, China

**Keywords:** polycystic ovary syndrome, PCOS, management, biomarker, microbiome, complication

## Introduction

Polycystic ovary syndrome (PCOS) is a complex endocrine disorder that affects a significant number of women of reproductive age worldwide (1, 2). It is characterized by a combination of clinical and biochemical features, including irregular menstrual cycles, hyperandrogenism, and polycystic ovaries (3). Beyond its reproductive manifestations, PCOS is often associated with various metabolic, reproductive, and psychological disturbances, making it a multifaceted condition with significant implications for women’s health.

Driven by the desire to understand its underlying mechanisms, identify diagnostic markers, and develop effective treatment strategies, PCOS has emerged as a topic of considerable interest within the medical and scientific community in recent years. Understanding the intricate mechanisms driving PCOS is vital for devising targeted interventions and improving clinical outcomes. More and more diseases are defined as PCOS-associated disorders, indicating its pathogenesis remains multifactorial. Efforts to manage PCOS encompass a comprehensive approach that addresses both the reproductive and metabolic aspects of the syndrome.

To discuss the challenges and future directions for PCOS, we call for the second round of paper in this Research Topic, providing an overview of the current understanding of PCOS mechanisms and highlight the advancements in its management. We anticipate that this topic will shed light on the complex nature of PCOS and emphasize the importance of a multidisciplinary approach to its diagnosis and treatment.

## Biomarkers of PCOS

Accurate and timely diagnosis of PCOS is paramount for effective management and prevention of associated complications. Nevertheless, the diagnostic process remains complex, hinging upon a combination of clinical evaluation, symptomatology, and laboratory investigations. An increasing focus has been placed on identifying and validating biomarkers that can assist in the diagnosis, classification, and prognosis of PCOS.

Guan et al. searched for the significantly different metabolites in follicle fluid and embryo culture medium, defining androsterone sulfate, glycerophosphocholine, and elaidic carnitine as potential biomarkers to predict the abortion rate of the PCOS group. Zhou et al. pointed out that the frequency of mucosal-associated invariant T cells was significantly reduced in the peripheral blood of PCOS patients. Furthermore, they also observed a corresponding higher level of the cytokine IL-17.

In reports of pioneering studies, it has been investigated that vitamin D deficiency may be involved in the pathophysiology of PCOS. Within our Research Topic, two articles specifically delve into the correlation between vitamin D and the syndrome. Białka-Kosiec et al. observed no correlation between the level of vitamin D and AMH, leptin, HOMA-IR and FGF23. Additionally, while the classical and alternate complement cascades were elevated in PCOS women, Moin et al. confirmed that they did not show a correlation with 1,25(OH)2D3. Both studies concluded that vitamin D may not serve as a reliable biomarker for PCOS.

## Management of PCOS

Therapeutic strategies for PCOS aim to alleviate symptoms, restore hormonal balance, improve fertility outcomes, and mitigate long-term health risks (4). Lifestyle modifications, including dietary changes, regular exercise, and weight management, form the cornerstone of non-pharmacological interventions. Pharmacological interventions, such as oral contraceptives, anti-androgens, and insulin-sensitizing agents, are often prescribed to manage specific symptoms and metabolic abnormalities.

For optimizing the outcomes of *in vitro* fertilization (IVF), Zeng et al. demonstrated the significance of adjusting the initial Gn dosage based on body weight to prevent ovarian hyperstimulation. Besides, a comparison implemented by Philbois et al. displayed no differences between moderate-intensity continuous training and high-intensity interval training group, thus suggesting both those training protocols were recommended for PCOS.

To gain insights into the updates of pharmacological interventions for PCOS, Xing et al. and Jiang et al. respectively recommended metformin plus liraglutide therapy and Cangfudaotan in improving reproductive abnormalities. Wang et al. provided an experimental study in a mouse model which depicts that the antibiotic cocktail intervention improved glucose metabolic disorders and hyperinsulinemia. The potential role for SGLT2 inhibitors was reviewed by Pruett et al. in treating obesity-associated cardiometabolic complications in PCOS.

## PCOS-associated diseases

Increasing evidence has supported a potential correlation between PCOS and asthma, which were previously regarded as diseases originating from two independent systems. A retrospective cross-sectional analysis by Juber et al. collected from February 2016 to April 2022 involving 1334 Emirati females revealed that pediatric asthma was an independent risk factor for adult PCOS. The view is strongly supported in the review by Xu et al., in which the correlation between asthma and PCOS highlighting the internal common pathophysiology and adverse influences on women’s health, is interpreted. Furthermore, given the shared common risk factors, Wu et al. presented the latest evidence of the bidirectional association between PCOS and periodontal disease. Wang et al. conducted a meta-analysis involving nine articles with 1,107 subjects and depicted that PCOS is positively associated with the risk of sleep disturbances.

Among the adverse impacts of PCOS, the adverse pregnancy outcomes shouldn’t be turned a blind eye on, apart from the impairment to woman herself. Emerging lines of evidence underscore the notion that PCOS has a negative impact on pregnancy outcomes, with an increased risk of gestational diabetes, hypertensive disease during pregnancy, and preterm birth (5, 6). In pregnancies affected by PCOS, the interrelated conditions are characterized by a dynamic interplay between hyperandrogenism and hyperinsulinemia. In the review summarized by Neven et al., the complex endocrine and metabolic interactions in pregnancies complicated by PCOS were elucidated.

## Concluding remarks

The growing prevalence of PCOS, along with its significant impact on women’s health, has prompted extensive research to unravel its underlying mechanisms and develop effective management strategies. The collection of papers that form part of this Research Topic displays the different facets of PCOS. We hope to provide investigators an extensive overview of cutting-edge issues in PCOS, further enriching their understanding of the mechanism reside with management of this complicated disorder.

## Author contributions

YSW drafted this editorial. PL, RL, YTW and HH revised and approved the final submitted version.
